# Recent advances in the role of mesenchymal stem cells as modulators in autoinflammatory diseases

**DOI:** 10.3389/fimmu.2024.1525380

**Published:** 2024-12-20

**Authors:** Baiyu Chen, Zhilei Chen, Mengfei He, Lijie Zhang, Longyan Yang, Lingling Wei

**Affiliations:** ^1^ Center for Endocrine Metabolism and Immune Diseases, Beijing Luhe Hospital, Capital Medical University, Beijing, China; ^2^ Beijing Key Laboratory of Diabetes Research and Care, Beijing, China

**Keywords:** mesenchymal stem cells, immunomodulation, autoimmune diseases, cell to cell contact, paracrine signaling, cell therapy

## Abstract

Mesenchymal stem cells (MSCs), recognized for their self-renewal and multi-lineage differentiation capabilities, have garnered considerable wide attention since their discovery in bone marrow. Recent studies have underscored the potential of MSCs in immune regulation, particularly in the context of autoimmune diseases, which arise from immune system imbalances and necessitate long-term treatment. Traditional immunosuppressive drugs, while effective, can lead to drug tolerance and adverse effects, including a heightened risk of infections and malignancies. Consequently, adjuvant therapy incorporating MSCs has emerged as a promising new treatment strategy, leveraging their immunomodulatory properties. This paper reviews the immunomodulatory mechanisms of MSCs and their application in autoimmune diseases, highlighting their potential to regulate immune responses and reduce inflammation. The immunomodulatory mechanisms of MSCs are primarily mediated through direct cell contact and paracrine activity with immune cells. This review lays the groundwork for the broader clinical application of MSCs in the future and underscores their significant scientific value and application prospects. Further research is expected to enhance the efficacy and safety of MSCs-based treatments for autoimmune diseases.

## 1 Introduction

Mesenchymal stem cells (MSCs), which are multipotent progenitor stromal cells, possess the remarkable potential of self-renewal and the ability to differentiate into multiple mesenchymal cell lineages. MSCs were first discovered in bone marrow by Friedenstein et al. and were later named mesenchymal stem cells by Caplan et al. (1–4). Pittenger et al. further discovered that adult stem cells could be induced to specifically differentiate into adipocytes, chondrocytes, or osteoblasts (5). And subsequent researchers found that MSCs could exist in various tissues such as adipose tissue, umbilical cord, and pulp, further highlighting their importance and potential therapeutic applications (6, 7). Besides possessing remarkable self-renewal capabilities, MSCs also exhibit extraordinary potential to differentiate into various cell lineages. One of the most notable features of MSCs is their capacity for self-renewal, enabling them to serve as promising candidates for cell and regenerative therapies (8, 9). Furthermore, these cells possess the versatility to differentiate into mesoderm lineages and, under specific conditions, even into ectoderm and endoderm tissues, showcasing their developmental plasticity (10). Importantly, studies indicate that MSCs exhibit a lower tumorigenesis risk due to their differentiation pathway typically avoiding malignant transformation, thereby alleviating concerns regarding the safety and side effects associated with stem cell therapies (11, 12). In recent years, extensive research has demonstrated that MSCs hold significant immunomodulatory potential (13–15). This makes them extremely promising candidates for the therapy of autoimmune inflammatory disorders. The immunomodulatory mechanisms of MSCs are primarily exerted through interactions with immune cells via direct cell contact and paracrine activity (16). Autoimmune disorders emerge as a result from an imbalance in the immune system that disturbs immunological tolerance (17). Most patients afflicted with autoimmune disorders require long-term, and in some cases even lifelong, therapies in order to maintain a basic quality of life. Some first-line drugs that primarily target interleukin-1 (IL-1), interleukin-6 (IL-6), and tumor necrosis factor-α (TNF-α) molecules have been shown to be beneficial in clinical treatment (18, 19). However, patients who use immunosuppressive drugs for long-term or high dosage may suffer from drug resistance and adverse reactions. These adverse reactions can include an increased risk of infection and the development of malignancy. Therefore, it is urgent to develop comprehensive treatment, combined with MSCs supplementary treatment. By doing so, we can harness the immunomodulatory potential of MSCs and achieve the crucial goal of controlling disease progression. Here, we review the immunomodulatory characteristics of MSCs and their recent applications in autoimmune diseases, so as to provide a basis for the practical application of MSCs.

## 2 Immunomodulation of MSCs

**Figure 1:**
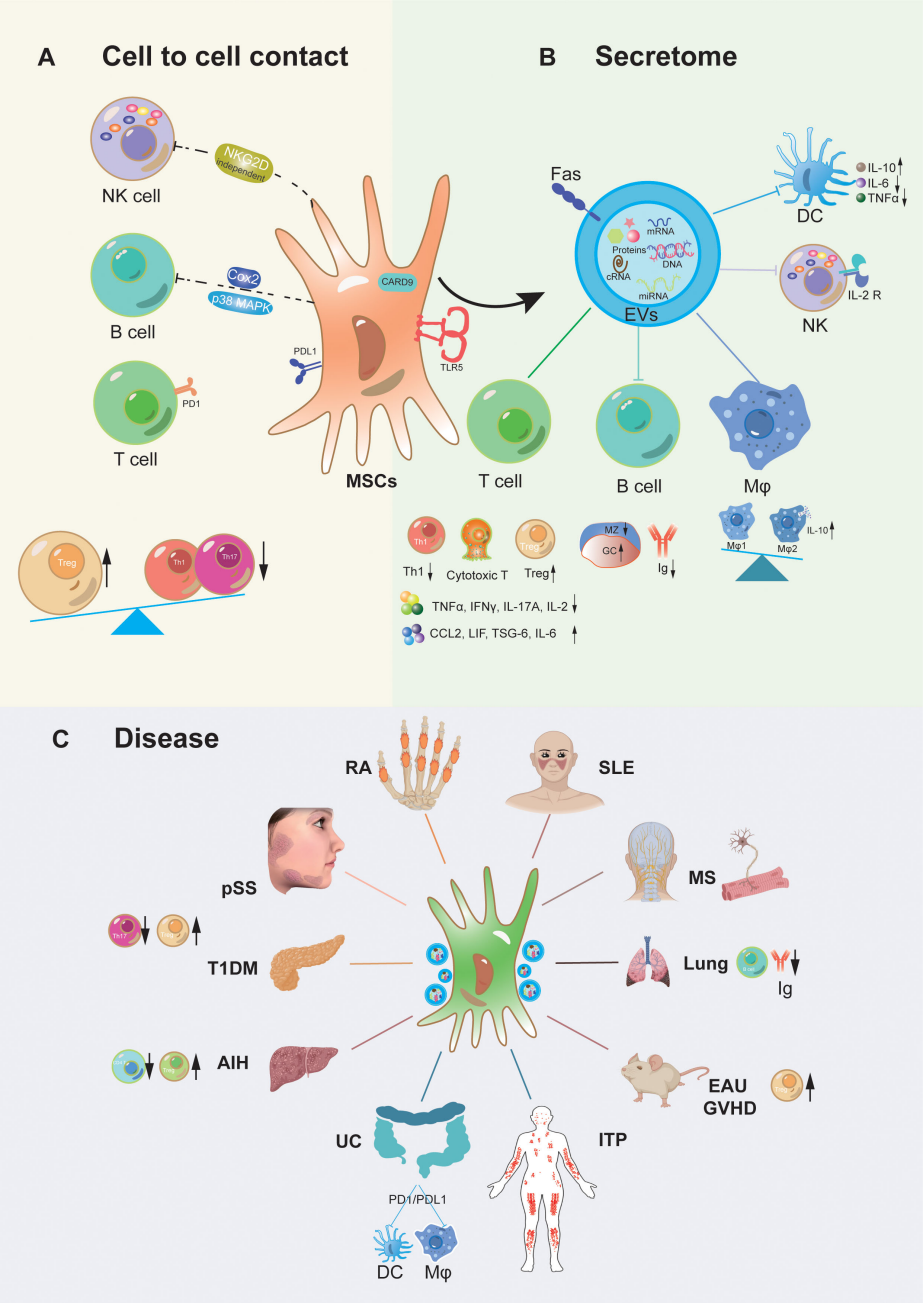
Mechanism of action of MSCs transplantation for autoinflammatory disorders. MSCs exert immunomodulatory functions primarily through direct interaction **(A)** with immune cells consist of T cells, B cells, and Nature killer (NK) cells etc., also via paracrine secretome (extracellular vesicles) **(B)** whose possession containing bioactive chemokines, cytokines, and growth factors, targeting on various immune cells, such as T cells, B cells, NK cells, Dendritic cells (DC), Macrophages (Mφ), etc. Meanwhile, MSCs derived from multiple tissues play pivotal roles in regulating immune cells and facilitating the potential therapy of associated inflammatory diseases **(C)**, such as Systemic lupus erythematosus (SLE), Mutiple sclerosis (MS), acute lung injury, primary sjogren syndrome (pSS), Rheumatoid arthritis (RA), Experimental autoimmune uveitis (EAU), Graft-versus-host disease (GVHD), Immune thrombocytopenia (ITP), Ulcerative colitis (UC), Autoimmune liver disease (AIH), Type 1 diabetic mellitus (T1DM), etc.

MSCs possess remarkable immunoregulatory properties that make them a subject of great interest in the field of immunology and regenerative medicine. MSCs participate in both innate and adaptive immune responses through direct cell contact and production of paracrine mediators. MSCs can directly interact with various immune cells, such as T cells, B cells, macrophages, natural killer cells (NKs), dendritic cells (DCs) and neutrophils (20, 21). Through these direct contacts, MSCs can modulate the activation, proliferation, and differentiation of immune cells. Additionally, MSCs secrete a variety of bioactive molecules, including cytokines, growth factors, and chemokines, which act in a paracrine manner to influence the behavior and function of immune cells. These paracrine factors can suppress the immune response, promote tissue repair, and regulate the inflammatory environment. The integration of direct cell contacts and paracrine activity equips MSCs with a potent immunomodulatory impact, allowing them to play a crucial role in treating autoimmune inflammatory diseases and various immune-related disorders. These include graft-versus-host disease (GVHD) (22–24), systemic lupus erythematosus (SLE) (25–27), psoriasis (28, 29), primary Sjögren’s syndrome (pSS) (30–32), type 1 diabetes mellitus (T1DM) (33–35), rheumatoid arthritis (RA) (36–38), among others (39–41). We summarized the mechanism of action of MSCs in the treatment of autoinflammatory diseases as shown in **Figure 1**.

### 2.1 Direct cell contacts with immune cells

One of the key immunoregulatory properties of MSCs is their ability to modulate the activity of immune cells. MSCs in a way that is dependent on direct cell-to-cell contact. This means that physical interaction between MSCs and immune cells is crucial for the immunomodulatory process. When MSCs come into contact with immune cells, they can exert their regulatory effects in several ways. For instance, they may modulate the activity of T cells. They can suppress the proliferation and activation of effector T cells, while promoting the generation and function of regulatory T cells. This helps to maintain immune balance and prevent excessive immune responses. MSCs can also interact with B cells. They may influence B cell differentiation, antibody production, and survival. By regulating B cell function, MSCs can contribute to the control of humoral immune responses. In addition, MSCs can have an impact on NKs. They may alter the cytotoxic activity and cytokine production of NKs, thereby modulating innate immune responses. Furthermore, MSCs may interact with DCs. They can affect DCs maturation, antigen presentation, and cytokine secretion. This can lead to changes in the initiation and regulation of adaptive immune responses. Overall, the direct cell contact-dependent mechanisms by which MSCs play an immunomodulatory function on immune cells are complex and multifaceted. These mechanisms play an important role in maintaining immune homeostasis and have potential applications in the treatment of various immune-mediated diseases.

#### 2.1.1 Immune regulation of T cells by MSCs

MSCs exhibit the expression of programmed cell death-ligand 1 (PD-L1), which engages with the programmed cell death-1 (PD-1) receptor on activated T cells. This interaction suppresses immunity by counterbalancing the activation signals of T cells. The PD-1/PD-L1 signaling pathway constitutes a vital immune checkpoint mechanism, safeguarding healthy tissues from immune attack, thereby playing a pivotal role in modulating immune responses and maintaining immune homeostasis (42, 43). Studies indicate that the expression of PD-1 ligands is upregulated in MSCs pretreated with interferon-gamma (IFN-γ), TNF-α, and interleukin-1 beta (IL-1β). Compared to unstimulated MSCs, the pretreated MSCs exhibit enhanced immunosuppressive effects on activated T cells and promote the differentiation of regulatory T cells (Tregs) in a PD-1-dependent manner. Notably, these effects can be reversed by introducing neutralizing antibodies against PD-L1 to MSCs, both *in vitro* and *in vivo* (44). A detailed analysis of single-cell transcriptomics and proteo-transcriptomics of MSCs isolated from various sources, including adipose tissue (AD), bone marrow (BM), placental villi (PM), and umbilical cord (UC), revealing that downregulation of PD-L1 impacts their immunosuppressive activity, which is regulated through GATA2 (45). In a NOD mouse model, MSCs mitigated the aggregation of T cells and macrophages in pancreatic islets induced by PD-L1/PD-1 blockade, thereby reducing the incidence of diabetes mellitus (46). When in direct cell-to-cell contact, allogeneic adipose-derived MSCs (ASCs) transfer active mitochondria and plasma membrane segments to Tregs, augmenting their immunosuppressive activity (47). Additionally, the transfer of MSCs mitochondria to activated CD4^+^ T cells results in the suppression of Th1 responses (48).

Overexpression of the C-C motif chemokine ligand 5 (CCL5) receptor C-C chemokine receptor 5 (CCR5) enhances the homing ability of MSCs and optimizes their immunomodulatory effects by decreasing infiltrating T cells and activated microglia, and inhibiting the activation of NLRP3 inflammatory vesicles in a mouse model designed to prevent classical experimental autoimmune uveitis (EAU) (49). Furthermore, direct and indirect interactions with allogeneic MSCs bolster Tregs’ capacity to accumulate immunosuppressive adenosine and inhibit the proliferation of conventional T cells. One direct exchange between Tregs and MSCs involves the transfer of active mitochondrial and plasma membrane fragments from MSCs to Tregs, a process that is HLA-dependent and correlated with a mismatch load of HLA-C and HLA-DRB1 eplet between Tregs and MSCs donors (47). Co-culture of MSCs derived from mouse inguinal fat with CD4^+^ T cells sorted from mouse spleen upregulated FcγRIIb expression on CD4^+^ T cells. Co-culture with sFgl2-MSCs reduced the proportion of Th17 and Th1 cells, increased the proportion of Tregs, and elevated the levels of phosphorylated SHP2 (p-SHP2) and phosphorylated SMAD2/3 (p-SMAD2/3) (50).

#### 2.1.2 Immunoregulation of MSCs to other immune cells

MSCs exert an influence on B cells through cell-dependent contact mechanisms. Research indicates that MSCs treated with IFN-γ inhibit the production of interleukin-10 (IL-10) by activated B cells via the Cox-2 pathway (51). Furthermore, MSCs arrest the B cell cycle in the G0/G1 phase, thereby inhibiting cell proliferation, by activating the p38 mitogen-activated protein kinase (MAPK) pathway (52). Another study showed that B cells and MSCs, when in contact, boosted VEGF production in MSCs. This increase then led to higher levels of pAKT and prevented caspase 3-mediated apoptosis in CD19^+^B cells (53).

In the context of innate immune responses, proinflammatory macrophages interact with MSCs, leading to an upregulation of CD200 expression on MSCs. This, in turn, facilitates the reprogramming of macrophages towards an anti-inflammatory phenotype through the binding of CD200 to CD200R on the surface of proinflammatory macrophages (54). Macrophages in ARDS models can enhance their energy metabolism and antibacterial capacity by phagocytosing mitochondria from MSCs, which are transferred through TNT-like structures (55). Additionally, MSCs can promote the differentiation of macrophages towards the M2 phenotype through PGE2,TSG-6,TGF-β,and so on Furthermore, MSCs promote the differentiation of macrophages into the M2 phenotype through factors like PGE2, TSG-6, and TGF-β (54, 56, 57).

Engineered MSCs with EACAM1 reduce NK cell activation and cytotoxicity through cell-to-cell interactions, regardless of NKG2D ligand regulation. MSCs can also inhibit T lymphocyte and NK cell function via HLA-G5 (58). Furthermore, MSCs can inhibit the differentiation of DCs through IL-6 (59). MSCs also release tumor necrosis factor-stimulated gene-6 protein (TSG-6) to suppress the expression of maturation markers on the surface of bone marrow-derived DCs, and reduce the ability of DCs to produce IL-12 and activate T cells (59). These engineered MSCs also effectively inhibit the proliferation of and activation T cells, as well as the inflammatory responses of monocytes (60). MSCs can temporarily reside in the lungs and are rapidly phagocytosed by monocytes, subsequently migrating to other parts of the body. After phagocytosing UCMSCs, monocytes polarize towards a non-classical phenotype, expressing PD-L1 and IL-10, while TNF-α levels decrease (61).

### 2.2 Function in paracrine activity

Considerable studies have shown that cytokines and exosomes secreted by MSCs can exert their actions on various immune cells via paracrine mechanisms to generate immunomodulatory effects, both in innate and adaptive immune responses. MSCs can also function independently of their cells themselves, but through the bioactive substances they secrete. MSCs-derived extracellular vesicles (MSC-EVs) not only have the same efficacy as MSCs (62, 63), but also have some advantages over parental fine MSCs due to their specific miRNA loading (64–69). MSC-EVs is not only easier to preserve, transfer, and produce, but also safer to administer, so it is receiving increasing attention (70).

#### 2.2.1 Immune regulation of T cells by MSC-EVs

Recently, research on the modulation of adaptive immunity by MSCs has primarily concentrated on their mechanisms of action on T cell populations, including the regulation of T cell proliferation and the differentiation of Th17 and Treg cells. One study revealed that *in vivo* administration of MSC-derived extracellular vesicles (MSC-EVs) significantly suppressed CD8^+^IFN-γ^+^ cytotoxic T cells (Tc1) and CD4^+^IFN-γ^+^ helper T cells (Th1), while decreasing the levels of pro-inflammatory cytokines TNF-α and IFN-γ. Additionally, it induced the generation of CD4^+^CD25^+^Foxp3^+^ Tregs and increased the levels of anti-inflammatory IL-10. *In vitro* experiments from the same study demonstrated that MSC-EVs also inhibited Tc1 and Th1 cells and promoted the induction of Tregs and related cytokines (71). In dental pulp MSCs (DPMSCs), amino acid deprivation (in humans) and nitric oxide (NO) production (in mice) have been shown to inhibit T cell proliferation within a specific concentration range. However, this inhibitory effect is restricted to local environments due to factors such as the short half-life of NO and the requirement for complete amino acid deprivation (72).

The lactate-dependent immunosuppressor mechanism was confirmed in UC-MSCs through the (delayed-type hypersensitivity, DTH) inflammatory model, and inhibition of lactate dehydrogenase (LDH) significantly reduced the ability of UC-MSCs to control the proliferation of activated CD4^+^ and CD8^+^ human T cells. Furthermore, high concentrations of L-lactic acid (25-50 mM) were found to profoundly affect the proliferation and differentiation of Th1 and Th17 cells (73). Research has demonstrated that exosomes derived from human umbilical cord mesenchymal stem cells (UCMSC-Exos) exhibit inhibitory effects on the abnormal proliferation and apoptosis of CD4^+^ T cells in individuals with pSS. These exosomes impede the G0/G1 cell cycle phase, prevent the progression into the S phase, diminish Th17 cell differentiation, and foster the differentiation of Tregs. Furthermore, UCMSC-Exos suppress the secretion of cytokines such as IFN-γ, TNF-α, IL-6, interleukin-17A (IL-17A), and IL-17F, while enhancing the production of IL-10 and TGF-β in pSS patients (74).

When Fas mutant mice were systemically supplemented with apoptotic vesicles (ApoVs), which directly interacted with CD4^+^ T cells, they inhibited CD25 expression and IL-2 production in a dose-dependent manner. The phosphatidylserine exposed on ApoVs mediated interactions with T cells, disrupting proximal T cell receptor signaling, reducing Th17 differentiation and memory T cell formation, and ameliorating inflammation and joint erosion in murine arthritis (75). Chen et al. prepared exosomes derived from human placental mesenchymal stem cells (hpMSC-ExoMSC), and RNAseq analysis demonstrated that Th17 differentiation was inhibited. Furthermore, hpMSC-ExoMSC improved the hypersecretory phenotype and cell-cell interactions in the hepatic Th17 microenvironment by modulating PERK/CHOP signaling (76). Using a mouse model of experimental autoimmune uveitis (EAU) and other *in vitro* assays, Kaur et al. demonstrated that MSC-EVs inhibit the MAPK/ERK pathway in activated T cells. Treatment with TGF-β1 or let-7b had a similar effect on the MAPK/ERK pathway (77). Cheung et al. analyzed the transcriptome of apoptotic MSCs and found that the ApoMSC secretome-dependent cyclooxygenase 2 (COX2)/prostaglandin E2 (PGE2) axis inhibited the proliferation and activation of human T cells and exerted chemo attractive effects on monocytes *in vitro*. Caspase activation under the regulation of the nuclear factor-kappa B (NF-κB) pathway induced upregulation of a range of immunosuppressive molecules in apoptotic MSCs, including COX2/PGE2, CCL2, LIF, TSG-6, and IL-6 (78). Ni et al. revealed that high expression of the CCL21-CCR7 axis in mice with autoimmune diseases promotes the targeted homing of MSCs to lymph nodes, thereby driving the specific distribution of MSCs. The results suggest that elevated levels of TNF-α, via the NF-κB pathway, induced transplanted MSCs to stimulate the secretion of L-amino acid oxidase (LAAO), which significantly inhibited Th17 cells. Additionally, indole-3-pyruvate (I3P) derived from LAAO acts as an effective inhibitor of Th17 cells by activating the aryl hydrocarbon receptor (AHR) pathway (79).

#### 2.2.2 Immunomodulatory effects of MSC-EVs on B cells

Currently, the precise mechanism by which MSCs regulate B cell immunity remains elusive. However, numerous *in vivo* and *in vitro* studies have revealed that MSCs can inhibit B cell proliferation and differentiation to a certain degree. Research conducted in CCL2 KO mice demonstrated that the absence of CCL2 augments B-cell receptor (BCR) signaling by upregulating the phosphorylation of the MST1-mTORC1-STAT1 axis. This leads to a reduction in marginal zone (MZ) B cells and an increase in germinal center (GC) B cells. *In vivo* inhibition of mTORC1 reverses the abnormal differentiation of MZ and GC B cells, and specific inhibitors *in vitro* can also rescue BCR signaling under antigenic stimulation. Additionally, the study found that CCL2 deficiency enhances early B-cell activation, including B-cell migration, clustering, and recruitment of signaling factors, via upregulation of the DOCK8-WASP-actin axis (80). Furthermore, another study involving lipopolysaccharide (LPS)-induced acute lung injury (ALI) mouse models suggested that MSCs may exert therapeutic effects on ALI by reducing the expression of chemokines associated with neutrophil recruitment and immunoglobulin production by lung B cells (81). In a separate study, olfactory extra mesenchymal stem cell-derived exosomes (Exos@SFMA) effectively alleviated synovial inflammation and joint destruction by significantly decreasing T follicular helper cell responses and further inhibiting GC B cell differentiation into plasma cells (39). Single-cell RNA sequencing has revealed that MSCs inhibit intrahepatic B-cell proliferation and cytokine production through exosomes, and regulate mitogen-activated protein kinase (MAPK) and nuclear NF-κB signaling pathways. These findings suggest that MSCs and their derived exosomes have diverse and promising therapeutic potential in modulating B cell immunity and related diseases (82).

#### 2.2.3 Immunomodulatory effects of MSC-EVs on macrophages

##### 2.2.3.1 MSCs promote the polarization of macrophages towards M2

MSCs possess the ability to regulate the activity and polarization of macrophages, thereby exerting immunosuppressive effects. In a LPS-induced abortion model, MSCs were found to promote the transition of decidual macrophages to the M2 phenotype in a manner dependent on tumor necrosis factor-stimulated gene-6 (TSG-6) (54). The research conducted by Zhuang et al. suggests that TSG-6 plays a pivotal role in regulating the immunomodulatory efficacy of MSCs’ mechanical response, which is modulated by the MAPK and Hippo signaling pathways and the downstream AP1 complex. This, in turn, influences macrophages through the CD44 receptor and inhibits the NF-κB pathway (83). Investigators discovered that MSCs primed with a TLR5 agonist (KMRC011) increased the secretion of immunosuppressive cytokines such as indoleamine 2,3-dioxygenase (IDO) and COX2, as well as the expression of M2 macrophage-polarizing cytokines like macrophage colony-stimulating factor (M-CSF) and IL-10 *in vitro*. These findings indicate enhanced immunosuppressive effects on lymphocyte proliferation. Furthermore, *in vivo* experiments demonstrated that KMRC011-induced MSCs ameliorated the severity of GVHD in a mouse model, with macrophages obtained from the spleens of mice showing a significant increase in the anti-inflammatory M2 phenotype (84). In a T1DM mouse model, hexyl 5-aminolevulinate (HAL)-loaded engineered cytokine-primed MSCs (H@TI-ev) exhibited high therapeutic efficacy by reducing CD4^+^ T cell density and activation through the PD-L1/PD-1 axis, and inducing macrophage transformation from M1 to M2 to remodel the immune microenvironment (85). Moreover, An et al. proposed a therapeutic strategy based on MSCs spheroids. In this strategy, local delivery of encapsulated MSCs spheroids *in vivo* in rats with myocardial infarction significantly attenuated local inflammation and subsequent fibrosis, while improving cardiac function by mediating the polarization of macrophages toward a healing-promoting M2 phenotype (86). These findings highlight the diverse and promising therapeutic potential of MSCs and their derivatives in modulating macrophage polarization and related diseases.

##### 2.2.3.2 MSCs inhibit macrophages

In recent years, an intriguing hypothesis has emerged among researchers, suggesting that lactate production serves as a crucial mediator of the immunosuppressive activity exhibited by MSCs (73). A separate study further illuminated this concept by demonstrating that MSCs, both *in vitro* and *in vivo*, effectively reduced the expression of CARD9, thereby inhibiting the phosphorylation of NF-κB in macrophages and preventing their polarization into the M1 phenotype (87). Moreover, adipose-derived stem cells (ASCs) have been shown to secrete STC-1, which negatively regulates the NLRC4 inflammasome in macrophages. This regulatory mechanism contributes to alleviating inflammation in lung tissue during pseudomonas aeruginosa (PA) infection (88). Additionally, allogeneic ASCs have been proven to play a protective role by enhancing macrophage recruitment and inducing their transition to the M2 phenotype through the HIF-1α/IL-10 signaling pathway (89). These findings collectively underscore the significant and multifaceted roles of MSCs and ASCs in modulating macrophage behavior and inflammation.

##### 2.2.3.3 MSCs interact with macrophages

Stimulation of human monocyte-derived macrophages (MDMs) with LPS or plasma samples from ARDS patients, categorized as either low-inflammatory or high-inflammatory phenotypes, in conjunction with treatment using mesenchymal stem cell-conditioned medium (CM) or extracellular vesicles (EVs), has led to the conclusion that MSCs regulate macrophages through the miR-181a-PTEN-pSTAT5-SOCS1 signaling axis (90). By altering the spatial distribution of MSCs and macrophages within a scaffold, the ‘cross-talk’ between these two cell types can be effectively manipulated, enabling controlled modulation of the scaffold-mediated bone immune response. In comparison to other multicellular models, the Taiji model, featuring a 2:1 ratio of MSCs to macrophages, demonstrated superior efficacy in activating anti-inflammatory M2 macrophages, enhancing osteogenic differentiation of MSCs, and accelerating new bone formation *in vivo*. This may be attributed to the activation of BMP-Smad, Oncostatin M (OSM), and Wnt/β-catenin signaling pathways in MSCs, mediated by macrophage-derived paracrine signaling factors (91). Wang et al. conducted indirect co-culture experiments with bone marrow-derived mesenchymal stem cells (BM-MSCs) inoculated on nanoporous titanium (Ti) scaffolds and macrophages. By blocking exosome secretion or extracting purified exosomes, they independently induced macrophage polarization. Their results indicated that, under the influence of TiO2 nanoporous topography, BM-MSCs can induce M1 polarization of macrophages, which may adversely affect the osteogenic microenvironment around implants (92). *In vitro* studies by Deng et al. further demonstrated that biotin-modified MSC-derived extracellular vesicles (Bio-EXs) are efficiently taken up by macrophages and exert immunomodulatory effects similar to those of unmodified MSC-EXs. Moreover, the Bio-GelMA@Bio-EX hydrogel sustained the release of MSC-EXs for seven days, promoting the polarization of macrophages towards the M2 phenotype (93). These findings collectively highlight the complex interplay between MSCs and macrophages in modulating the bone immune response and provide insights into the potential therapeutic applications of MSC-derived extracellular vesicles and hydrogels in bone regeneration and immune modulation.

#### 2.2.4 Immunomodulatory effects of MSC-EVs on DCs

Dendritic cells (DCs) are antigen-presenting cells that fall within the category of innate immune cells. They possess the capability to detect antigenic substances from the external environment and present them on their cell surface to T cells. The immunoregulatory effects of MSCs on DCs are primarily characterized by inhibiting the differentiation, maturation, and antigen presentation of DCs, as well as reducing the expression of pro-inflammatory factors (94). In a model of T1DM, MSCs have been found to induce the secretion of IL-10 by immature DCs *in vitro*, thereby intercepting the initiation and expansion of autoreactive T cells in tissue inflammation (95). Furthermore, in streptozotocin (STZ)-induced diabetic mice, extracellular vesicles derived from mouse adipose-derived mesenchymal stem cells (mADSC-EVs) significantly diminished the inflammatory response involving DCs. These vesicles were also observed to continuously upregulate the protein expression of the NGF/TrkA pathway and decrease theLPS-mediated expression of IL-6 and TNF-α (96). These findings suggest that MSCs and their derived extracellular vesicles play a crucial role in modulating the immune response, particularly in the context of autoimmune diseases such as T1DM. By targeting DCs, MSCs and their derivatives may offer promising therapeutic strategies for the treatment of these conditions.

#### 2.2.5 Immunomodulatory effects of MSC-EVs on NKs

Natural killer cells (NKs) constitute a vital component of the innate immune system, providing swift responses to virus-infected and tumorigenic cells (97). MSCs exhibit the capacity to modulate NK cell activity by inhibiting their proliferation, disrupting their cytotoxicity, and suppressing the secretion of proinflammatory cytokines. Investigations into the effects of soluble factors derived from adipose-derived mesenchymal stem cell (ADSC) media, collectively termed the ADSC secretome, on NK cells have revealed intriguing findings. Specifically, the presence of ADSC secretome was found to attenuate IL-2 signaling in IL-2-activated NK cells by inhibiting the phosphorylation of JAK1 and JAK3 kinases. Concurrently, the induction of CIS (cytokine-inducible Src homology 2 domain-containing protein) and DUSP4 (dual-specificity phosphatase 4) was enhanced (98). These results suggest that the ADSC secretome plays a pivotal role in modulating NK cell activity through the regulation of IL-2 signaling and the induction of CIS and DUSP4. This modulation may have important implications for the development of therapeutic strategies targeting NK cell-mediated immune responses in various diseases.

## 3 Immunomodulatory role of MSCs in autoimmune inflammatory diseases

Traditional treatments for autoimmune inflammatory diseases commonly involve the administration of immunosuppressive drugs, corticosteroids, and nonsteroidal anti-inflammatory drugs. These medications are designed to quell the hyperactive immune response and mitigate inflammation. However, they frequently entail adverse side effects and may not yield satisfactory results in all patients. MSCs have emerged as a promising therapeutic alternative for autoimmune inflammatory diseases, thanks to their immunomodulatory capabilities. The integration of conventional therapies with MSCs presents a hopeful strategy for addressing autoimmune inflammatory diseases. Conventional treatments can swiftly manage inflammation and alleviate symptoms, whereas MSCs can offer sustained immunomodulation and facilitate tissue repair. By merging these two methodologies, it may be feasible to attain superior treatment outcomes and decrease the reliance on high doses of immunosuppressive drugs. We summarized the completed and ongoing clinical trials of MSCs in the treatment of autoinflammatory diseases in **Table 1**. The results of these registered studies all demonstrate the safety and efficacy of MSCs in the treatment of autoimmune diseases.

**Table 1:** Registered clinical trials of MSCs for the treatment of autoimmune diseases.

Inflammatory diseases	Trial number	Sponsor	Status	Phase	MSCs source	Participant Group /Arm
Multiple Sclerosis	NCT00395200	University of Cambridge, UK	completed	phase I/II	Autologous BM-MSCs	MSCs, i.v.
	NCT00781872	Hadassah Medical Organization, Israel	completed	phase I/II	Autologous BM-MSCs	Autologous BM-MSCs, i.t. Autologous BM-MSCs, i.v.
	NCT01377870	Royan Institute, Tehran, Iran	completed	phase I/II	Autologous BM-MSCs	MSCs, i.v. Placebo, i.v.
	NCT01606215	Imperial College London, UK	completed	phase I/II	Autologous BM-MSCs	MSCs, i.v. Placebo, i.v.
	NCT01730547	Karolinska Institute, Sweden	completed	phase I/II	Autologous BM-MSCs	Autologous BM-MSCs, i.v.
	NCT02166021	Dimitrios Karussis, Hadassah Medical Organization (Responsible Party), Israel	completed	phase II	Autologous BM-MSCs	MSCs, i.t. MSCs, i.v. Placebo, i.v.
	NCT01895439	University of Jordan, Jordan	completed	phase I/II	BM-MSCs	Autologous BM-MSCs, i.v.
	NCT03326505	University of Jordan, Jordan	completed	phase I/II	Allogenic UC-MSCs	UC-MSCs, intrathecal UC- MSCs and SPT, intrathecal Supervised Physical Therapy (SPT)
	NCT05116540	Hope Biosciences Stem Cell Research Foundation, US	completed	phase II	Autologous HB-MSC	MSCs, i.v. Placebo, i.v.
	NCT03355365	Tisch Multiple Sclerosis Research Center of New York, US	completed	phase II/III	Autologous MSC-NP	MSC-NP, i.t.
	NCT00813969	The Cleveland Clinic, US	completed	phase I	Autologous MSCs	MSCs transplantation
	NCT04823000	Hadassah Medical Organization, Israel	completed	phase I/II	Autologous MSCs	MSCs, i.t. MSCs, i.v.
	NCT02239393	Ottawa Hospital Research Institute, Canada	completed	phase II	MSCs	MSCs, i.v. Placebo, i.v.
	NCT04749667	Haukeland University Hospital, Bergen, Norway	Active, not recruiting	phase I/II	Autologous BM-MSCs	Crossover with MSCs at baseline and placebo at 6 months Crossover with placebo at baseline and MSCs at 6 months
	NCT04956744	ImStem Biotechnology, Inc. Farmington, US	recruiting	phase I	hESC-MSCs	Low Dose, i.v. High Dose, i.v. Optional Dose, i.v.
	NCT02587715	Novo Cellular Medicine Institute LLP, India	unknown	phase I/II	Allogeneic UC-MSCs	MSCs Liberation therapy
	NCT01854957	Antonio Uccelli, University of Genova (Responsible Party), Italy	unknown	phase I/II	Autologous MSCs	MSCs, i.v. Placebo, i.v.
Type 1 Diabetes Mellitus	NCT00690066	Mesoblast, Inc. New York, US	completed	phase II	MSCs	PROCHYMAL®Placebo, i.v.
	NCT04061746	Medical University of South Carolina, US	Recruiting	phase I	Allogeneic UC-MSCs	Allogeneic UC-MSCs,i.v. Placebo,i.v.
	NCT03484741	Van Hanh General Hospital, HCMC, Vietnam	unknown	phase I/II	Allogeneic UC-MSCs	MSCs and PRP, i.v.
	NCT04078308	Royan Institute, Tehran, Iran	unknown	phase I/II	Autologous BM-MSCs	MSCs, i.v. Placebo, i.v.
	NCT00646724	Fuzhou General Hospital, China	unknown	phase I/II	Autologous MSCs	Cotransplantation of islet and MSCs
Rheumatoid Arthritis	NCT03691909	Hope Biosciences, Texas, US	completed	phase I/II	Autologous HB-MSC	Autologous HB-MSC, i.v.
	NCT01873625	Royan Institute, Tehran, Iran	completed	phase II	BM-MSCs	MSCs / Placebo
	NCT01547091	Alliancells Bioscience Corporation Limited, China	unknown	phase I/II	UC-MSCs	UC-MSCs,i.v. Rheumatoid Arthritis with Disease Modifying Drugs (DMARDs) UC-MSC+DMARDS,i.v.
	NCT03798028	Xijing Hospital, China	Unknown	NA	UC-MSCs	UC-MSCs,i.v. no UC-MSCs,i.v.
Systemic Lupus Erythematosus	NCT03171194	Medical University of South Carolina, US	completed	phase I	Allogeneic UC-MSCs	MSCs i.v.
	NCT05631717	The Affiliated Nanjing Drum Tower Hospital of Nanjing University Medical School, China	Recruiting	phase III	hUC-MSCs	hUC-MSCs, i.v. IL-2, s.c.
	NCT03917797	Universidad de los Andes, Chile	Recruiting	phase II	UC-MSCs	MSCs, i.v. Standard of Care, i.v. Placebo, i.v.
Sjogren’ s syndrome	NCT06392711	University of Wisconsin, Madison, US	not yet recruiting	phase I	BM-MSCs	MSCs Dose Level 0 into one submandibular gland MSCs into both submandibular glands-Dose Escalation Cohort MSCs into both submandibular glands - Expansion Cohort
Immune thrombocytopenia	NCT04014166	Institute of Hematology & Blood Diseases Hospital, China	Active, not recruiting	NA	hUC-MSCs	Low Dose hUC-MSCs,i.v. Medium Dose hUC-MSCs,i.v. High Dose hUC-MSCs,i.v.

N/A, not available; Intravenous Injections, i.v.; Intrathecal injection, i.t.; subcutaneous injection, s.c.

### 3.1 Cell to cell contact with immune cells

#### 3.1.1 Immune regulation of T cells by MSCs in autoimmune diseases

Currently, the utilization of PD-L1 inhibitors in autoimmune diseases is still in its nascent research phase. A deeper understanding of the mechanism and suitable conditions for PD-L1 inhibitors in various autoimmune diseases is imperative, and further clinical trials are essential to validate their efficacy and safety.

Several animal studies have delved into the potential of mesenchymal stem cells (MSCs) in treating various autoinflammatory diseases. For example, MSCs with high PD-L1 expression have demonstrated improvements in hyperglycemia and obesity in STZ-induced T1DM mice (85). Additionally, MSCs derived from decidual teeth have been found to restore the Treg and Th17 cell balance via the PD-L1/PD-1 pathway, leading to reduced glandular inflammation and alleviation of dry symptoms in mice with Sjögren’s syndrome (99). In models of graft-versus-host disease (GVHD), MSC treatment has been shown to lower clinical scores and extend survival in mice, with these benefits being reversed by PD-L1-specific antibodies both *in vivo* and *in vitro* (44). In a mouse model of classical experimental autoimmune uveitis (EAU), Yuan et al. discovered that overexpression of CCR5, a CCL5 receptor, in MSCs enhanced their homing ability and improved their immunomodulatory effects in preventing EAU by decreasing the number of infiltrating T cells and activated microglial cells, inhibiting the activation of the Nlrp3 inflammasome, and improving their immunomodulatory effect in preventing EAU (49). Wei et al. administered 1×10^6^ hUC-MSCs derived from umbilical cord tissue intravenously to mice with autoimmune hepatitis (AIH) and assessed liver function and inflammation by measuring serum levels of alanine aminotransferase and aspartate aminotransferase, as well as pathological damage to liver tissue. They observed a significant decrease in CD4^+^ T-cell infiltration in the liver and a reduction in the frequency of IFNγ- and IL-17A-producing CD4^+^ T-cells in the spleen, along with a trend towards an increase in Treg cells in liver tissue. RNA sequencing analysis of liver tissues revealed significant negative regulation of inflammation-related signaling pathways in the UC-MSC-treated group, suggesting that hUC-MSCs have the potential to suppress the immune response and provide a potential clinical option for ameliorating AIH (100). Wu et al. found that infusion of TGFBI-knockout hUC-MSCs had an impaired therapeutic effect on T1DM mice, increasing T-cell infiltration and the expression of IFN-γ and IL-17A in the spleen. They also discovered that TGFBI derived from hUC-MSCs inhibited the proliferation of activated T cells by interfering with the expression of the G1/S checkpoint CyclinD2, suggesting that TGFBI may be a new target for T1DM therapy (101).

#### 3.1.2 Immunoregulation of MSCs to other immune cells in autoimmune diseases

Furthermore, the application of human adipose-derived MSCs has the potential to diminish the infiltration and accumulation of T cells and CXCL9^+^ macrophages within the islet beta cell area (46). Moreover, MSCs engineered to express CEACAM1 have demonstrated efficacy in ameliorating inflammatory manifestations and enhancing survival rates in GVHD mouse models, achieved by mitigating NK cell activation and cytotoxicity, as well as suppressing T cell proliferative activation (60).

### 3.2 Immunomodulatory function by paracrine activity

In preclinical studies involving mouse models of dextran sulfate sodium (DSC)-induced ulcerative colitis (UC) and imiquimod (IMQ)-induced psoriasis, the researchers demonstrated that mesenchymal stem cell-derived small extracellular vesicles (MSC-sEVs) expressing PD-L1 effectively inhibited inflammatory immune cells, targeting and repairing tissue damage through the PD-1/PD-L1 pathway. The results of this assay revealed that MSC-sEVs-PD-L1 suppressed the proliferation of T cells, dendritic cells, and macrophages, leading to a reconfiguration of the local immune microenvironment via the induction of regulatory T cells and the elimination of effector T cells, accompanied by modulation of cytokine expression (102). Given its straightforward preparation, low cost, feasibility, and biosafety, the researchers believe this technique holds promising potential for clinical application. In the realm of GVHD, extracellular vesicles derived from mesenchymal stem cells (MEXs) have been shown to alleviate clinical symptoms and extend the survival of recipient mice by promoting the differentiation of Treg cells in the spleen while preserving the cytotoxic anti-leukemia effect of recipient mouse CD8^+^ T cells (94). Compared to the mechanism of intercellular contact, the paracrine mechanism of MSCs has been extensively studied across various clinical, animal, and cellular research studies, particularly focusing on the regulation of T cells and macrophages. Based on multicellular organ tissues established in Mdr2^-/-^ mice and patients with primary sclerosing cholangitis (PSC), Chen et al. proposed a potential therapeutic role for exosomes derived from human placenta-derived mesenchymal stem cells (ExoMSC) in hepatic fibrosis associated with PSC or Th17-related diseases. Animal studies showed that ExoMSC ameliorated hepatic fibrosis in Mdr2^-/-^ mice, significantly reducing collagen deposition in the precatheteric region, inhibiting Th17 differentiation, and decreasing the proportion of CD4^+^IL-17A^+^ T cells *in vivo*. Multicellular organ tissue studies further revealed that ExoMSC alleviated the hypersecretory phenotype and cell-cell interactions in the hepatic Th17 microenvironment by modulating PERK/CHOP signaling (76).

#### 3.2.1 Immune regulation of T cells by MSCs in autoimmune diseases

A study conducted *in vivo* revealed that a dexamethasone-based liposomal integrated MSCs (Dexlip-MSCs) treatment approach significantly extends the drug’s circulation time in the body and reduces the necessary dosage compared to dexamethasone alone. Dexlip-MSCs were found to upregulate the expression of CRISPLD2 and other anti-inflammatory factors by activating the glucocorticoid receptor signaling pathway. This led to a decrease in pro-inflammatory factors and an enhanced anti-inflammatory inhibitory effect on CD4^+^ T cells, ultimately mitigating the progression of systemic lupus erythematosus (SLE) in MRL/lpr mice (25). In a separate clinical trial involving 30 patients with conventionally refractory active SLE, umbilical cord-derived mesenchymal stem cells (UCMSCs) transfusions were administered. The results showed an upregulation of Treg cell expression and a downregulation of Th17 expression through TGF-β and PGE2, effectively alleviating the clinical symptoms of SLE patients. These findings suggest a promising new approach for MSCs as adjuvant therapy for SLE (103). However, in the NCT01539902 clinical trial indicates that hUC-MSC has no significant additional effects beyond standard immunosuppression. Eighteen patients with WHO classification III or IV lupus nephritis (LN) were randomly assigned to hUC-MSCs (2×10^8^ cells) or placebo. All patients received standard immunosuppressive therapy, including intravenous methylprednisolone and cyclophosphamide, followed by oral prednisolone and mortemycophenolate. Nine of 12 patients (75%) in the hUC-MSC group experienced a response, compared with five of six patients (83%) in the placebo group. The proportion of patients achieving a complete response was similar in both groups, along with similar improvements in serum albumin, complement, kidney function, systemic lupus erythematosus disease activity index and the British Isles Lupus Assessment Group score. The trial was abandoned after enrolling 18 patients when it became clear it would not show a positive therapeutic effect (104). Li et al. conducted trial NCT01741857 and found that circulating miR-320b and MAP3K1 may be involved in the proliferation of SLE CD4^+^ T cells. Small RNA sequencing analysis revealed a significant decrease in circulating miR-320b levels in SLE patients after mesenchymal stem cell transplantation (MSCT). *In vitro* experiments further showed that reduced levels of MAP3K1 in SLE peripheral blood mononuclear cells (PBMCs) were associated with CD4^+^ T cell proliferation. In MRL/Lpr mice, miR-320b overexpression exacerbated SLE symptoms, while inhibition of miR-320b promoted disease remission (105).

In the NCT01941394 clinical trial, patients undergoing allogeneic hematopoietic stem cell transplantation were randomized into two groups: one receiving standard GVHD prophylaxis and the other also receiving MSC infusion when leukocytes recovered to 1,000 cells/μL (transplantation, day E0). After 30 days, patients injected with MSCs showed significantly increased CD4^+^ T-cell counts, as well as increased levels of IL-6, IL-8, IL-17, TNF-α, and IFN-γ. The concentrations of G-CSF, GM-CSF, PDGFbb, FGFb, and IL-5 also increased on day E+30. Regardless of MSC administration, there was a significant increase in the number of CD8^+^ cells at day E+30, while the concentrations of IL-9, eotaxin, IP-10, MCP-1, and MIP-1a were all increased at day 30. These results suggest a positive effect of MSC administration on the recovery of T-cell subsets and the immune system in patients after allogeneic HSCT (106). García-Berna et al. conducted a controlled HCELL-MSC/HCELL^+^ MSCs administration assay using a mouse model of acute graft-versus-host disease (aGVHD). The results suggested that MSC surface HCELL/CD44 connections significantly enhanced the immunomodulatory activity of MSCs by inducing the secretion of multiple potent immunomodulatory molecules, including IL-10 (107).The efficacy and safety of umbilical cord mesenchymal stem cells was demonstrated in a phase 2 study to prevent chronic graft-versus-host disease after haploid hematopoietic stem cell transplantation. The results suggest that repeated MSCs infusion may suppress aGVHD symptoms in HLA-haplo HSCT patients, accompanied by changes in T, B, and NK cell numbers and subtypes, resulting in immune tolerance (108). In psoriasis, co-culture of DPMSCs with CD3^+^ T cells demonstrated inhibitory effects on T cell proliferation and induction of apoptosis. DMSCs also decreased the Th17/Treg cell ratio and enhanced Treg-mediated inhibition of effector T cells. These effects were partially attenuated by TGF-β receptor inhibitors, indicating a potential therapeutic strategy for ameliorating psoriasis symptoms (48). Furthermore, sFgl2 gene-modified MSCs were found to promote Treg cell differentiation, inhibit Th17 and Th1 cell differentiation, and exhibit a strong therapeutic effect in an experimental model of autoimmune hepatitis induced by con A (50). UCMSC-derived exosomes were shown to reduce elevated levels of autophagy in peripheral blood CD4^+^ T cells of patients with primary Sjögren’s syndrome. These exosomes regulated T cell proliferation and apoptosis, inhibited the differentiation of Th17 cells, and promoted the differentiation of Treg cells, ultimately restoring the Th17/Treg cell balance in these patients (74). *In vitro* studies have shown that TLR3 stimulation can specifically enhance the immunosuppressive effects of MSCs on the proliferation and differentiation of Th1 and Th17 subtypes, while TLR4 agonists can completely reverse these effects (109, 110). Kaur et al. compared MSC-EV generated from monolayer cultures (ML-EV) or microcarrier cultures (MC-EV) using a mouse model of experimental autoimmune uveitis (EAU) and other *in vitro* assays reflecting the *in vivo* mode of action of MSC-EV. The results showed that MC-EV, carrying a high concentration of TGF-β1 EV, had greater efficacy in blocking disease progression, inducing apoptosis, and inhibiting retinal-reactive T cell chemotaxis in EAU mice compared to ML-EV. They also proposed a mechanism by which MSC-EV, as well as treatment with TGF-β1 or let-7b, could inhibit the MAPK/ERK pathway of activated T cells (77). Wang et al. found that human umbilical cord mesenchymal stem cell-derived exosomes (hUCMSCs-EVs) could alleviate dry eye disease (DED) symptoms by multi-targeting the IRAK1/TAB2/NF-κB pathway through certain miRNAs. In the DED model induced by a dry environment combined with scopolamine administration, treatment with hUCMSC-EVs increased tear volume and maintained corneal integrity in DED mice. It also decreased the levels of proinflammatory cytokines in tears, increased the density of microglandular vesicle cells, and inhibited apoptosis and CD4^+^ cell infiltration (111). Chen et al. compared intestinal Peyer’s patches (PP)-derived MSCs with bone marrow-derived MSCs and systematically demonstrated the prominent benefits of mouse MSC proteins in the treatment of inflammatory bowel disease (IBD) in mice, partly through IL-22 (112). Related clinical studies have demonstrated the safety of MSCs therapy. Specifically, a phase I clinical trial involving Cymerus mesenchymal stem cells (CYP-001) was conducted at seven centers in the UK and Australia to address steroid-resistant aGVHD (SR-aGVHD) in adults post-allogeneic hematopoietic stem cell transplantation. Participants were stratified into two groups: Group A (n=8) received two intravenous infusions of CYP-001, one on day 0 and one on day 7, at a dose of 1×10^6^ cells per kilogram of body weight, capping at a maximum absolute dose of 1×10^8^ cells. Group B (n=7) similarly received infusions on days 0 and 7, but at an escalated dose of 2×10^6^ cells per kilogram, with a maximum absolute dose of 2×10^8^ cells. Notably, no participants received any other investigational drugs within 28 days of prior to the first CYP-001 dose. Reporting the 2-year follow-up results, we found that 9 out of 15 participants (60%) survived, a figure more favorable than those reported in previous SR-aGVHD studies. The causes of death were complications commonly observed in allogeneic hematopoietic stem cell transplant recipients and were deemed unrelated to CYP-001 treatment by the investigators. Furthermore, no serious adverse events, tumors, or other safety concerns associated with CYP-001 were reported (113).

#### 3.2.2 Immunoregulation of MSCs to other immune cells in autoimmune diseases

Ranjbar and colleagues conducted a Phase I trial involving nine patients with biopsy-confirmed, standard therapy-refractory LN. They administered a systemic infusion of 2×10^6^ allogeneic adipose-derived MSCs (AD-MSCs) per kilogram of body weight and monitored the patients for 12 months. The follow-up results indicated that allogeneic AD-MSC transplantation exhibited a favorable safety profile and was effective in decreasing urinary protein excretion and reducing disease activity. Notably, the most significant improvements in proteinuria and disease activity scores were observed at 1 and 6 months post-intervention (114). Furthermore, research has delved into the immunomodulatory mechanisms of MSCs, particularly their ability to regulate macrophages. The administration of human umbilical cord-derived MSC-derived extracellular vesicles (hUC-MSCs-EVs) induced macrophages to adopt an anti-inflammatory M2 phenotype. These induced M2 macrophages demonstrated a robust capacity to inhibit CD4^+^ T cell proliferation and promote the generation of Tregs *in vitro*, thereby reducing inflammation and mitigating tissue damage. Additionally, MSCs pretreated with the TLR5 agonist KMRC011 exhibited enhanced immunosuppressive effects on lymphocyte proliferation in both *in vivo* and *in vitro* mouse GVHD models. This pretreatment increased the secretion of immunosuppressive cytokines such as IDO and COX2, and enhanced the expression of M2 macrophage polarization cytokines, including M-CSF and IL-10 *in vitro*. This treatment facilitated the polarization of macrophages towards the M2 phenotype, thereby increasing the proportion of anti-inflammatory cells and alleviating GVHD severity in mice. These findings present a novel perspective for GVHD treatment (84). Moreover, studies have emphasized the significance of MHCI on MSCs in NK cell-mediated killing and immunosuppression (115). MEXs have been shown to inhibit the expression of costimulatory molecules and the secretion of cytokines derived from DCs (94). Exos@SFMA have proven effective in alleviating synovial inflammation and joint destruction by markedly reducing the responsiveness of follicular helper T cells and further inhibiting the differentiation of germinal center B cells into plasma cells (39). A randomized Phase 2 trial conducted at 15 sites in nine countries tested the safety and activity of MSC in the treatment of multiple sclerosis. 144 patients with active relapsing-remitting or progressive multiple sclerosis were randomly assigned according to a cross-over design, with one group (n=69) receiving a single intravenous injection of autologous bone marrow-derived MSCs followed by placebo at week 24 and the other group (n=75) receiving placebo at week 24 followed by autologous MSCs. They were followed up at 48 weeks. 213 adverse events were recorded, again distributed between groups (93 were recorded in 35 of 69 patients who received MSC for the first time, compared with 120 in 42 of 75 patients who received placebo for the first time). The most commonly reported adverse events were infection and infestations, with 54 of 213 adverse events (18 of 93 in the early MSCs group and 36 of 120 in the late MSCs group). Nine serious adverse events were reported among seven patients in the placebo group, while none were reported in the MSC group. All serious adverse events were considered unrelated to the therapeutic infusion. No deaths were reported during the study period. However, no effect was shown on GEL (an MRI surrogate marker for acute inflammation) in patients with active MS at week 24, so the conclusions of this study do not support the use of bone marrow-derived MSCS in the treatment of active MS (116).

## 4 Discussion

MSCs exhibit the potential to significantly enhance the effectiveness of conventional therapies by mitigating drug resistance and alleviating adverse side effects. Their ability to promote the regeneration of damaged tissues further underscores their importance, as it can lead to improved functional recovery and enhanced quality of life for patients.

### 4.1 Immunomodulatory characteristics of MSCs

MSCs exert their immunomodulatory effects through direct cell contact with immune cells, such as T cells, B cells, macrophages, and dendritic cells, as well as through the secretion of paracrine mediators. This dual mode of action allows MSCs to regulate the activation, proliferation, and differentiation of immune cells, thereby modulating the immune response. The production of cytokines, growth factors, and chemokines by MSCs further enhances their immunomodulatory capacity, promoting tissue repair and regulating the inflammatory environment.

MSCs significantly impact T cell activity by suppressing activation and proliferation through the PD-1/PD-L1 pathway, an immune checkpoint mechanism. Pretreatment with inflammatory cytokines enhances MSCs’ immunosuppressive effects, suggesting dynamic immunomodulation. MSCs also regulate B cell function by inhibiting proliferation and antibody production, affecting IL-10 production and B cell cycle progression. They interact with innate immune cells, reprogramming macrophages and influencing NK cell activation and cytotoxicity. MSCs affect DCs maturation and function, impacting both innate and adaptive immune responses. These findings highlight MSCs’ potential as an immunotherapeutic tool, but further research is needed to fully understand the mechanisms and explore clinical applications.

The paracrine activity of MSCs has emerged as a pivotal mechanism through which these cells exert their immunomodulatory effects on various immune cell populations. By secreting cytokines and exosomes, MSCs can influence both innate and adaptive immune responses, making them a promising therapeutic tool in modulating immune dysfunction. The studies reviewed provide compelling evidence that MSC-EVs regulate T cell proliferation and differentiation, suppressing Tc and Th1 cells while enhancing Tregs and anti-inflammatory IL-10 (71, 75, 89, 117). MSC-EVs also regulate T cell signaling pathways, such as inhibiting the MAPK/ERK pathway and modulating PERK/CHOP signaling (77). This shift towards a tolerogenic immune environment suggests a therapeutic role in autoimmune and inflammatory disorders. MSCs and their EVs have multifaceted immunomodulatory effects on macrophages, DCs, and NK cells, offering therapeutic potential in autoimmune diseases and cancer immunotherapy. Future research should focus on elucidating mechanisms and exploring therapeutic applications. Understanding the mechanisms of MSCs-mediated immune modulation will facilitate the development of targeted therapeutic strategies. MSCs and their EVs, especially PD-L1-expressing sEVs, show promise in preclinical and clinical studies for treating inflammatory and autoimmune diseases.

### 4.2 Exploration of application in animal model

In mouse models of UC and psoriasis, MSC-sEVs-PD-L1 suppressed immune cell proliferation, induced Tregs, and modulated cytokines, facilitating tissue repair. MSCs’ paracrine mechanism, studied extensively, also helps regulate T cells and macrophages, with MEXs alleviating GVHD symptoms in mice (94). Exosomes from placenta-derived MSCs ameliorate hepatic fibrosis in animal studies. In SLE, MSCs and their derivatives show variable results as adjuvant therapy, possibly due to patient differences. MicroRNA modulation, like miR-320b in SLE CD4^+^ T cells, suggests novel therapeutic strategies (105). MSCs also aid immune reconstitution after HSCT and enhance immunomodulatory activity in GVHD mouse models. Further clinical trials are needed to validate these therapies.

### 4.3 Clinical trials and emerging trends

A multitude of clinical trials have been initiated to rigorously assess the safety and efficacy of MSCs in the management of autoimmune inflammatory diseases. These trials have yielded encouraging results, demonstrating that MSCs can ameliorate symptoms, diminish disease activity, and elevate patients’ overall quality of life. The studies discussed highlight the therapeutic potential and safety of MSCs and their derivatives in treating immune-mediated diseases. DPMSCs show efficacy in preventing GVHD, psoriasis, and autoimmune diseases by modulating immune cell populations and T cell differentiation (89, 118, 119). Gene-modified MSCs and exosomes also exhibit therapeutic effects. Research explores enhancing MSCs’ immunosuppressive effects through TLR stimulation and different culture methods for generating MSC-EVs. MC-EVs show promise in blocking disease progression and alleviating symptoms A phase I trial supports the safety of MSCs in treating GVHD. Ranjbar’s study demonstrates the safety and efficacy of allogeneic AD-MSCs in reducing LN symptoms, with sustained therapeutic effects (114). Huang et al. conducted an open-label, multicenter, parallel randomized clinical trial to evaluate the efficacy of repeated umbilical cord MSCs infusions during the early post-transplant period (days 45 and 100) for preventing chronic graft-versus-host disease (cGVHD) after haploidentical hematopoietic stem cell transplantation (haplo-HSCT). The results of this trial reveal that early repeated MSCs infusions decrease the incidence and severity of cGVHD and enhance graft-versus-host disease-free and relapse-free survival (GRFS) among patients, without raising the incidence of adverse events (120). A total of 203 patients with steroid-resistant acute graft-versus-host disease (SR aGVHD) from nine centers in China took part in a multicenter, randomized, open-label phase 3 clinical trial to evaluate the efficacy and safety of MSCs combined with basiliximab and calcineurin inhibitors as second-line therapy for SR aGVHD. The trial results demonstrate that the combination of MSCs with second-line treatment improves therapeutic outcomes, reduces drug toxicity and the incidence of cGVHD, without increasing the risk of relapse, and is well-tolerated, highlighting the promising therapeutic potential of MSC-based combination therapy (121). The immunomodulatory mechanisms MSCs, especially their capacity to regulate macrophages, are of interest. Pretreating of MSCs with a TLR5 agonist intensifies their immunosuppressive effects, representing a promising strategy for GVHD treatment (84). The significance of MHC I on MSCs in NK cell-mediated killing and immunosuppression is also noted (115). Exos@SFMA alleviates synovial inflammation in arthritis models (39). However, a phase 2 trial in multiple sclerosis showed mixed results, indicating that further research is required (116).

Despite these promising findings, there remains a pressing need for additional research to refine the integration of MSCs with conventional therapies. Future endeavors should concentrate on pinpointing the most suitable cell source, determining the optimal dosage, and establishing the most effective route of administration for MSCs. Equally important is the continued evaluation of the long-term safety and sustained efficacy of this innovative therapeutic approach.

In summation, the synergistic combination of conventional therapies and MSCs stands as an auspicious strategy in the treatment of autoimmune inflammatory diseases. This paradigm shift holds the potential to elevate treatment outcomes and alleviate the considerable burden these diseases impose on patients. As we advance, it is imperative to delve deeper into the intricate mechanisms of action of MSCs and to optimize their utilization within the clinical setting, ultimately paving the way for more targeted and effective therapeutic interventions. Overall, MSCs offer promising therapeutic options for immune-mediated diseases, with immune modulation and tolerance induction as potential mechanisms. Further research and larger trials are required to confirm efficacy and safety in different patient populations and disease contexts.
